# Evaluation of the Ability of Seven Active Ingredients of Fungicides to Suppress *Phytophthora cactorum* at Diverse Life Stages, and Variability in Resistance Found among Isolates

**DOI:** 10.3390/jof8101039

**Published:** 2022-09-30

**Authors:** Asad Ali, Ram Kumar, Jana Mazákova, Marie Maňasová, Miloslav Zouhar, Matěj Pánek

**Affiliations:** 1 Department of Plant Protection, Faculty of Agrobiology, Food and Natural Resources, Czech University of Life Sciences Prague, Kamýcká 129, 165 00 Prague, Czech Republic; 2 Team of Ecology and Diagnostics of Fungal Plant Pathogens, Crop Research Institute, Drnovská 507/73, 161 06 Prague, Czech Republic

**Keywords:** *Phytophthora cactorum*, fungicide resistance, mycelial growth inhibition

## Abstract

*Phytophthora cactorum* is considered an important plant pathogen which is causing major damage to strawberry plants worldwide. In the current study, the ability of the active ingredients of seven different fungicides, azoxystrobin, cymoxanil, dimethomorph, fenamidone, fluopicolide, metalaxyl and propamocarb, to suppress the mycelial growth, sporangial formation and zoospore release of *P. cactorum* isolates, was tested. The variation in resistance against various fungicides was found among the isolates. The active ingredients are also unequally efficient against different life stages of *P. cactorum*, which is probably associated with their different modes of action. A significant level of resistance was recorded against metalaxyl and dimethomorph; however, these were totally inefficient against the zoospore release, while azoxystrobin did not inhibit mycelial growth. The only fungicide efficient against all three *P. cactorum* life stages tested was fluopicolide, although the calculated resistance factor gives evidence of the rise of resistance in the majority of isolates even against this fungicide. Significant differences were found between responses to fungicides of isolates from strawberry and from other host species. Based on the Mahalanobis distances calculated in the discriminant analysis comprising all of the assays performed, the similarities among isolates were estimated.

## 1. Introduction

*Phytophthora cactorum (Lebert et Cohn) Schroet.*, a fungus-like organism from the family Peronosporaceae with a cosmopolitan distribution, is one of the most important plant pathogens [1]. In 1870, Lebert and Cohn in Central Europe first discovered *P. cactorum* on cacti *Carnegiea gigantea* (Engelm). Britton and Rose discovered it on *Melocactus nigrotomentosus* [2]. This pathogen has a wide host range, including more than 200 plant species [3]. This species poses a serious danger to plants and trees in ornamental horticulture, and has the potential to decrease the production of many economically important crops such as apples, pears and strawberries, causing rotting of the roots, crowns and collars, and fruit infections [4]. In strawberries, *P. cactorum* is responsible for crown rot and leather rot which is associated with major economic losses for the agricultural sector [5]. As this pathogen is one of the major limiting factors in the commercial strawberry production system worldwide [6], this has instigated an effort to restrict its distribution, spread and incidence in the fields. 

The exact origin and natural distribution of many *Phytophthora* spp. is difficult to assess because the human-caused global movement of plants, including those asymptomatically infected, has distributed these pathogen species worldwide [7,8]. For example, *P. nicotianae* was reported for the first time in Indonesia in 1895 [9]; in the USA it was described in 1915 [3], but its presence in European greenhouses has been confirmed since 1927 [10]. Goss et al. [11] described *P. ramorum* migrations between Europe, the USA and Canada, but the unclear origin and spread of this pathogen still remains under investigation. The worldwide migration and importance of *P. infestans* is also notorious [12]. 

The first disease incidence of leather rot caused by *P. cactorum* was reported in the United States in 1924 [13]; other cases were gradually reported in Europe and also in Asia, while the first description of crown rot was reported in 1952 in Germany [14], in 1988 in Sweden [15], in 1990 in Finland [16] and in 1992 in Norway [17]. Although the widespread distribution of this pathogen in Europe was reported by the aforementioned researchers, *P. cactorum* is considered an alien species to the European continent, which is evidenced by its low genetic variability, close phylogenetic relatedness to other non-native species, and high virulence to host plant species which are indigenous to Europe [1,18].

The combination of cultural practices and chemical control methods used to be considered an efficient way to reduce the disease incidence rate [19,20]. Fungicides like mefenoxam, azoxystrobin, pyraclostrobin [21] and dimethomorph [22] were most effective in controlling *P. cactorum* infections, but soon after their commercial release, the development of a resistance to fungicides became a serious problem [23]. The rise of resistance depends on the frequency and dose of applications of fungicides [24]. The resistance of *P. cactorum* against metalaxyl was first reported in 1988 by Utkhede and Gupta in an in vitro environment [25], while the resistance of *P. cactorum* isolates to mefenoxam in strawberry fields in the USA was reported for the first time by Jeffers et al. in 2004 [26]. Recently, Marin and Peres [27] reported that the fungicides cymoxanil, fluopicolide, mandipropamid and oxathiapiprolin completely inhibited the mycelial growth of *P. cactorum* isolates from Florida at a concentration of 1 µg/mL, but significant differences in the resistance to some chemical fungicides have already been found between *P. cactorum* isolates or their genetic lineages [28,29,30,31,32,33,34]. 

*P. cactorum* strains have been reported as host-specific by various researchers. Van Der Scheer [32], Harris and Stickels [33] and Hantula et al. [4] tested the infection potential of *P. cactorum* isolated from different hosts to cause crown rot in strawberries, and they concluded that crown rot can only be caused by isolates, isolated from strawberry crowns. Seemüller and Schmidle [34] found that *P. cactorum* strains isolated from strawberry crowns were less pathogenic on apple bark tissue, and vice versa. They also mentioned that *P. cactorum* isolates from any other host are capable of causing leather rot in strawberry fruit, but not crown rot. Similar conclusions were reported by Belisario et al. [35], Oudemans and Coffey [36] and Cooke et al. [37]. The more detailed population structure of *P. cactorum* was described by Pánek et al. [38], differentiating four genetic lineages of a complex of *P. cactorum* based on isolates originating from woody plants. Recently, isolates from strawberry plants from central Europe were described as another distant genetic lineage [39], also confirming the previous conclusions of lineage differentiation.

The current study was designed to evaluate the sensitivity pattern of *P. cactorum* isolates from different hosts, distinguishing strawberry and non-strawberry isolates. In the current study, the effects of seven active ingredients of fungicides with a different mode of action, i.e., azoxystrobin, cymoxanil, dimethomorph, fluopicolide, fenamidone, metalaxyl and propamocarb, on the mycelial growth inhibition of *P. cactorum* isolates, were evaluated. Four of these fungicides were evaluated as effective and have subsequently been chosen for tests of inhibition of sporangial development and the release of zoospores from sporangia. Additionally, we compared the relations between isolates based on their response to fungicides to relations based on their genetic distances published earlier [39].

## 2. Materials and Methods

### 2.1. Acquisition and Maintenance of Isolates

Twenty-one *P. cactorum* isolates originating from strawberry plants in the Czech Republic were used, completed by nine isolates from woody host plants. The isolates were differentiated into two groups: ‘S’, originating from strawberry plants, and ‘W’, originating from woody hosts. The details of isolates used in this study are given in Table 1. The members of the W group represented three genetic lineages, according to Pánek et al. [38,39]. The isolates were cultured on V8 juice agar (V8A; commercial V8 juice, 200 mL; CaCO_3_, 4 g; agar 16 g; distilled water 800 mL; autoclaved for 60 min at 121 °C) Petri dishes at 25 °C for seven days and preserved at 4 °C for further use in the following experiments.

### 2.2. In-Vitro Mycelial Growth Inhibition Assay

The active ingredients of seven fungicides, i.e., azoxystrobin, cymoxanil, dimethomorph, fluopicolide, fenamidone, metalaxyl and propamocarb, were obtained from Sigma-Aldrich, Germany, in an analytical grade with more than 98% purity. For the preparation of a stock solution with a concentration of 10 mg/mL, 100 mg of active ingredients were dissolved in 10 mL of analytical grade dimethyl sulfoxide (DMSO) with ≥99.5% purity (obtained from Sigma-Aldrich, St. Louis, MO, USA) and stored at 4 °C in the dark to preserve their fungicidal potential before their use in the particular experiments. The active ingredients of fungicides and their concentrations used for particular assays in this study are given in Table 2.

To establish the growth test, the mycelial plugs were cut from the margins of actively growing colonies developed on V8 agar plates, using a 6 mm cork-borer, and placed inverted in the center of a 90 mm V8 agar in 90 mm Petri dishes amended by the tested fungicides in desirable concentrations (Table 2). In the control plates, no fungicide was added. After incubation at 25 °C for 8 days in the dark, the mycelial growth was measured in two orthogonal directions using a digital Vernier caliper. The daily growth was expressed after subtracting the inoculating agar plug diameter from the total diameter of the colony; the value was then divided by two to acquire the radius dimension, which was subsequently divided by the number of days of cultivation to acquire the value of daily growth. The mycelial growth inhibition percentage was expressed by relating the growth on the amended plate to the growth on the non-amended control, using the method mentioned by Yahyazadeh et al. [40]. This assay was performed in triplicates for all isolates and concentrations; the average of these three measurements was used for statistical analyses.

### 2.3. Sporangia Formation Assay

Azoxystrobin, dimethomorph, fluopicolide and metalaxyl were used in the assay of inhibition of sporangia formation. This assay was performed as reported by Matheron and Porchas [30], with slight modifications. Agar plugs of a 6 mm diameter, cut from the margins of actively growing *P. cactorum* colonies, were transferred to 90 mm Petri dishes and submerged in 20 mL of sterile distilled water, amended by the tested fungicides in the respective concentrations (Table 2). After incubation at 25 °C for 48 h under a 10 h photoperiod, the mycelial plugs were transferred to Eppendorf tubes and crushed, followed by the addition of 1 ml of liquid from the respective Petri dish. The content of the Eppendorf tubes was vortexed for 2 minutes at 500 rpm, and the number of sporangia was then counted using a Bürker hemocytometer counting chamber [41]. In total, nine repetitions were performed for each combination of isolate and concentration of the respective fungicide. In the control experiment, no active ingredient of fungicide was added to the sterile distilled water. 

### 2.4. Zoospore Discharge Assay

Azoxystrobin, dimethomorph, fluopicolide and metalaxyl were used for the assay of zoospore discharge inhibition. This assay was performed according to a somewhat modified protocol developed by Matheron and Porchas [30]. Sporangia were produced by transferring 6 mm mycelial plugs of the growing *P. cactorum* colony to Petri dishes containing 20 ml of sterile distilled water. After incubation at 25 °C for 48 h, the distilled water in the Petri dishes was decanted, and 20 mL of sterile distilled water amended with active ingredients of fungicides of desired concentrations (Table 2) was added. All the dishes were cooled down at 4 °C for 2 h. After such treatment, the Petri dishes were incubated at 25 °C for 2 days under a 10 h photoperiod for the zoospore release. The number of empty sporangia was counted by a Bürker hemocytometer counting chamber, as mentioned by Gunetti et al. [41]. Each combination of isolate and the particular concentration of the respective fungicide was repeated nine times. For the control experiment, 20 m of sterile distilled water was used without the addition of any fungicide.

### 2.5. Data Analysis

**The determination of EC_50_:** The EC_50_ values were calculated for each fungicide used in the mycelial growth inhibition assay, as well as the sporangia formation and zoospore release assay, using the GraphPad Prism software (9.0.0) for Windows OS (GraphPad Software, San Diego, CA, USA), according to a protocol described by Li et al. [42]. To convert the heterogenous variance of the data to homogenous, the data of the growth values for all fungicide concentrations tested against each isolate was transformed into logarithmic form, followed by the normalization of all of the data measured. Such normalization was performed after defining the plateaus in the data with respect to the inhibition response, i.e., the maximum inhibition in the experiment, induced by the highest fungicide concentration, was defined as a 100% response of isolates, and no inhibition at zero concentration was considered a 0% response of isolates. After the normalization of the data, a nonlinear regression curve fit model was applied and the EC_50_ values were calculated. For fluopicolide, the theoretical values of the resistance factor (RF) [43] were calculated in an effort to objectivize the resistance level against this fungicide. The RF expresses the ratio between the EC_50_ of the resistant isolate and the EC_50_ of the sensitive one, optimally a member of a wild population. Since the values of EC_50_ for a wild *P. cactorum* population were not available, we estimated the RF values by comparing the highest and the lowest fluopicolide EC_50_ that we calculated for *P. cactorum* to the highest and lowest values calculated for a wild population of another *Phytophthora* spp.: *P. capsici* 0.11–0.16 µg/mL [43], *P. nicotianae* 0.06–0.15 µg/mL [44] and *P. erythroseptica* 0.08–0.35 [45].

**Kruskal–Wallis tests (K-W):** Using the environment of the software Statistica 13.0 (TIBCO Software Inc., Palo Alto, CA, USA), the differences between isolates or their groups on different levels were evaluated by the nonparametric Kruskal–Wallis test. Basically, the significance of the differences between isolates originating from strawberry (S) and those from woody plants (W) was evaluated. In case we discovered that some isolates (i.e., highly resistant or sensitive isolates) had a sharply different reaction to any fungicide, the significance of their difference to other isolates was also tested; in such case these isolates were assembled into a new group to enable us to test their differences against the standard S and W groups. The significance of the differences was evaluated considering the inhibition rates at all concentrations of a single fungicide together and on the level of concentrations of each fungicide. This comparison was performed for all three assays, i.e., the mycelial growth inhibition assay, inhibition of sporangial production assay and inhibition of zoospore release assay.

**Discriminant analysis (DA):** This analysis was performed to confirm the reasonability of differentiating between considered isolate groups based on more than one evaluated variable (i.e., all concentrations of each fungicide together; all fungicides together; and all three assays together), and to find the concentration of each fungicide in the respective assay (growth inhibition rate, inhibition of sporangial production, inhibition of zoospore release) that was most important for discrimination between the considered isolate groups. In this analysis, the membership of isolates to groups according to their original host (strawberry—S, woody plants—W) was used as a grouping variable. These analyses resulted in values for total Wilks λ (λ_Wtot_), which express the ability of the model to discriminate between the groups considered (0 = the best discrimination, 1 = the worst discrimination) based on the measured values of the dependent variable (i.e., the inhibition rates at all concentrations of the respective fungicide, or of all fungicides together). The values of partial λ_W_ associated with each of the considered dependent variables (the inhibition rate by a particular fungicide, or by its particular concentrations) expressed the importance of these variables in differentiating between the considered groups. The values of λ_Wtot_ and partial λ_W_ also indirectly express the variability of the effect of each fungicide against various *P. cactorum* isolates. The canonical part of the discriminant analysis resulted from the calculation of standardized canonical roots and the expression of the relative contribution of each dependent variable (i.e., fungicides, or their particular concentrations) to these roots. The roots with a significant ability to express differences between the considered groups were depicted graphically by scatter charts. Additionally, discriminant analysis based on the data for inhibition rates in all analyses (i.e., the growth inhibition, sporangial production inhibition and zoospore release inhibition at all concentrations and for all fungicides tested) resulted in a matrix of Mahalanobis distances between isolates. 

**Comparison of genetic and phenotypic data:** For twenty-two isolates used both in the current study and in a population analysis based on the ddRADseq published before [39], the comparison of resulting similarity matrices was enabled. To create the similarity matrix based on all fungicidal inhibition rates, the Mahalanobis distances calculated in discriminant analysis (DA) were converted, where the distance value 0.000 was considered a 100% similarity between isolates, while other distances were recalculated to a percentage in relation to the highest total distance found in the whole Mahalanobis distances matrix. The concordance between the similarity matrix created in this way and the ddRADseq similarity matrix was expressed in a percentage; thus, 100% meant absolute concordance and a zero value meant no similarity.

## 3. Results

### 3.1. In-Vitro Mycelial Growth Inhibition Assay

From the seven tested fungicides—metalaxyl, dimethomorph, fluopicolide, azoxystrobin, propamocarb, cymoxanil and fenamidone—only the first three have a significant effect on the mycelial growth inhibition of *P. cactorum* isolates when cultivated on amended V8 Petri dishes. Although metalaxyl, dimethomorph and fluopicolide were confirmed as being effective against the majority of the tested isolates, important differences were found between the levels of inhibition. The EC_50_ values for each isolate and fungicide are given in Table 3, while growth inhibition rates are available in supplementary Table S1.

**Figure 1:**
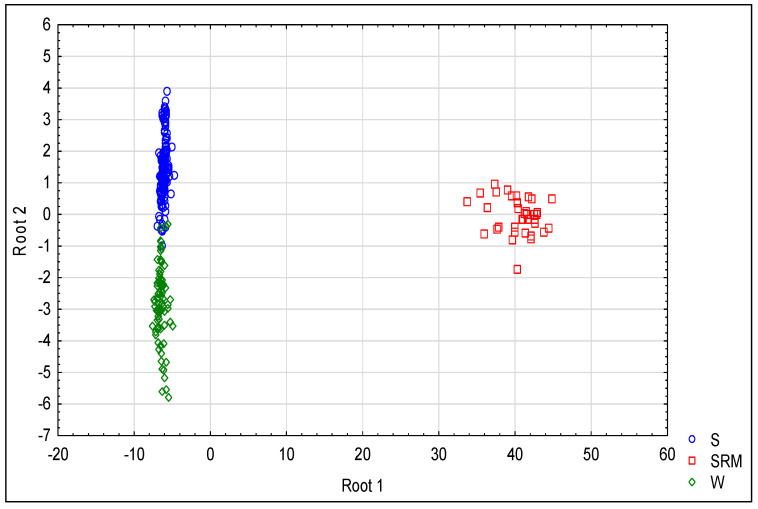
Similarities between the responses of *P. cactorum* isolates to metalaxyl in the mycelial growth inhibition assay. The results of canonical discriminant analysis depict the differences according to canonical root 1 as only being associated with resistance to metalaxyl, and does not differentiate groups W and S. The differences according to root 2 depict the component of variability of resistance associated with membership in these two groups. The grouping used: S—isolates from strawberry plants, SRM—isolates from strawberry plants resistant to metalaxyl and W—isolates from woody plants.

The mycelial growth of the majority of the *P. cactorum* isolates, i.e., 26 out of 30, was almost 100% inhibited by metalaxyl at concentrations between 20.00 and 60.00 µg/mL, with EC_50_ ranging from 0.033 to 1.993 µg/mL. The remaining four isolates, originating from strawberry plants, were found to be resistant against metalaxyl, with EC_50_ values greater than 4,000,000 µg/mL. The growth of these isolates was not significantly inhibited even at the highest tested concentration of 100 µg/mL. Although significant differences were found between resistant and non-resistant isolates (Table 4), the differences between groups, defined according to their original host plant species (W and S groups), were confirmed as significant by the K–W test at only two medium concentration levels. The differences in the inhibition rates at those two concentrations were as high as 25% and 5%. The discriminant analysis based on all of the concentrations, however, differentiates well between these groups, which is obvious from the value of λ_W_ = 0.256 (*p* = 0.001; Table 5) and is clearly depicted in Figure 1. The revealed modes of resistance seem to have only two states: absolute sensitivity and absolute resistance. Resistance against this fungicide seems to be most probably the property of individual isolates but not the property of their group.

**Figure 2:**
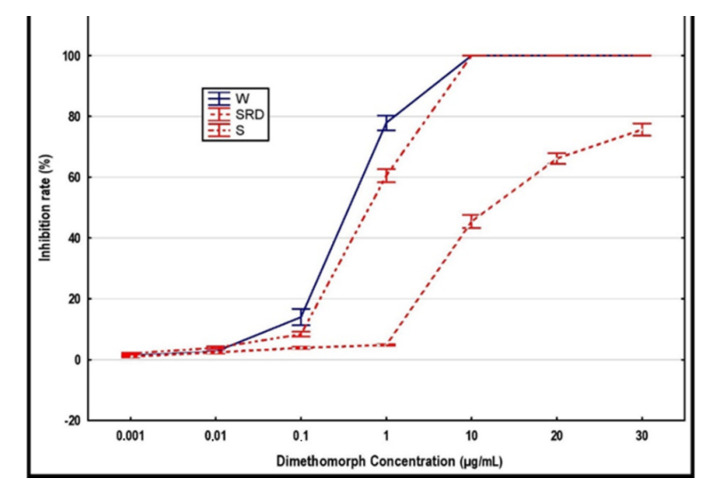
All tested isolates were organized into groups according to the host plant they were isolated from: S—strawberry, W—woody hosts. The group marked as SRD was created by isolates originating from strawberry plants which are obviously resistant to dimethomorph. The values of the mean and 95% confidence intervals are depicted.

Dimethomorph was efficient in mycelial growth inhibition because the majority of isolates, i.e., 27 out of 30, were classified as dimethomorph-sensitive. Their mycelial growth was inhibited 100% at the concentration of 10 µg/mL, where the EC_50_ ranged from 0.13 to 1.29 µg/mL. Only three isolates from the S group were evaluated as resistant against dimethomorph, with EC_50_ values from 11.32 to 12.52 µg/mL (Table 3). The inhibition rate of these resistant isolates gradually increased with the increasing concentration of dimethomorph, and reached about 75% at 30 µg/mL (Table 6 ). Two of these three isolates were simultaneously resistant against metalaxyl. These three isolates created a distant group, which differed from both the S and W groups at all concentration levels higher than 0.1 µg/mL (Figure 2). The resistance of groups S and W also mutually differed at concentration levels up to 1.0 µg/mL. (Table 6).

Another fungicide evaluated as efficient against *P. cactorum* isolates was fluopicolide, which inhibited the mycelial growth of all *P. cactorum* isolates at 20 µg/mL. A significant exception was created by two isolates originating from woody plants, whose growth was already 100% inhibited at 10 µg/mL. At this concentration, their inhibition rate was more than 20% higher than that of the remaining isolates, thus they could be considered to be more sensitive against this fungicide (Table 7). A comparison of the S and W groups resulted in the conclusion that they are significantly different at the concentration level of 0.10 and higher. Although the differences in the values of the average inhibition rate were not very high, the W group seems to be a few percent more inhibited by this fungicide. If the two more highly sensitive isolates (which originated from woody plants) were considered as a separate group, the differences between the S and W groups were evaluated mainly as insignificant, and only these two sensitive isolates differed significantly (*p* = 0.000) from both the S and W groups at concentration levels higher than 0.10 µg/mL (Table 7). The EC_50_ values for fluopicolide range from 0.7048 to 3.999 µg/mL (Table 3). The estimations of the resistance factor (RF), calculated for fluopicolide as the rate of the EC_50_ of the *P. cactorum* isolates to the EC_50_ of the members of some wild populations of *Phytophthora* spp. (*P. capsica, P. nicotianae* and *P. erythroseptica*) [43,44,45], were of a magnitude between 2.0 and 66.7. Such magnitudes imply that all *P. cactorum* isolates included in the study have an intermediate resistance [43].

Although the remaining fungicides, i.e., azoxystrobin, propamocarb, cymoxanil and fenamidone, were evaluated as ineffective in the mycelial growth inhibition of the *P. cactorum* isolates even at the highest concentrations tested, significant differences in inhibition rates between the S and W groups were found for some of them (Table 5). The inhibition rate of cymoxanil and fenamidone for the W group isolates was higher than for the S group, although the differences were small. The differences at the highest concentration, i.e., 100 µg/mL, were insignificant. Details of the mycelial inhibition assay are given in supplementary Table S1.

### 3.2. Sporangia Formation Assay

The formation of zoosporangia is considerably influenced by the presence of metalaxyl in the majority of isolates at the concentration of 10.0 µg/mL, with a mean EC_50_ value of 0.683 µg/mL. The four S group isolates which were revealed to be resistant in the growth inhibition assay were also confirmed as resistant in the sporangial production assay, with a recorded EC_50_ value >270,000 µg/mL (Table 3). Their sporangial production was not influenced at all tested concentrations, thus they differed significantly from both remaining S group isolates as well as from the W group. The remaining S isolates were more sensitive to metalaxyl in medium concentrations (0.10 and 1.00 µg/mL) than the W group isolates; the highest concentration was similarly effective against all isolates except for these resistant isolates (supplementary Table S3). Details of the percentage inhibition of the sporangia formation assay are given in supplementary Table S2.

Dimethomorph was efficient at inhibiting sporangia formation, with EC_50_ ranges from 0.0769 to 1.78 µg/mL, including three isolates from the S group resistant to dimethomorph in the mycelial growth inhibition assay. Additionally, the sporangial formation of these three isolates was completely inhibited, and their EC_50_ values were less than 1.00 µg/mL. The sporangial inhibition was recorded from 95% to 100% at a concentration of 20 µg/mL of dimethomorph, and no significant differences were found between the S and W groups (K-W test, *p* = 0.7923). No significant differences were found, even if the S group isolates resistant against this fungicide in the growth inhibition assay were considered as a separate group, in addition to the standard S and W groups (K-W test, *p* = 0.8970).

Fluopicolide was evaluated as efficient against all tested *P. cactorum* isolates, whose sporangial production was 100% inhibited at the concentration 10.0 µg/mL. No obviously resistant isolates were found, and no differences in sensitivity were revealed between the S and W groups on the level of all concentrations tested together (K-W test, p = 0.197). Significant differences were found at medium concentrations (0.1 and 1.0 µg/mL); the W group was inhibited ca. 20% higher than the S group at the first mentioned concentration (supplementary Table S4). The example of fluopicolide shows that medium concentrations most accentuated the differences between isolates, whereas low concentrations were not effective at all, and high concentrations affected all of the isolates. Despite these differences, this fungicide was confirmed as the most consistent in inhibiting sporangial production, with the recorded EC_50_ ranging from 0.106 to 0.633 µg/mL (Table 3).

Relatively high differences between isolates of *P. cactorum* were discovered for azoxystrobin on the level of both of the isolates and their groups, W and S. Nevertheless, at least five isolates can be considered resistant against this fungicide, reaching an inhibition rate of ca. 15–80% even at the highest concentration tested, 10.0 µg/mL, while the inhibition of the remaining isolates was close to 100%. No sharp border between resistant and not-resistant isolates was found. In addition, there were considerable differences between the groups S and W on the level of single concentrations (K-W test, *p* < 0.000; supplementary Table S5), where the S group isolates were already inhibited more distinctively at concentrations of 0.01 µg/mL. The difference was most significant at 10.00 µg/mL, reaching a rate as high as 10%, although both the S and W groups contain resistant isolates. The differences between resistant and non-resistant isolates are also obvious from the results of DA analysis, where λ_W_ = 0.258 (*p* < 0.000) indicates a good differentiation between them. In such a differentiation, all concentrations above the level of 0.1 µg/mL participated, and membership in the resistant isolate groups seemed to be more important than membership in the S and W groups. The EC_50_ of all isolates recorded for the sporangia formation assay ranged from 0.038 to 9.429 µg/mL except for one isolate from the W group which displayed a high EC_50_ value, namely 65,628 µg/mL (Table 3). 

### 3.3. Zoospore Discharge Assay

The efficiency of metalaxyl against zoospore release is low; the maximum inhibition was as low as ca. 17% at a concentration of 100 µg/mL. The differences in sensitivity between the isolates are insignificant. On the level of the isolate groups S and W, there are statistically significant differences (as low as ca. 2%), however, at concentrations higher than 1.00 µg/mL according to the Kruskal–Wallis test (*p* = 0.023) given in supplementary Table S6. Such structuring was not confirmed by the DA based on all of the concentrations. The differences between isolates or their groups in sensitivity against metalaxyl should be considered unimportant (Table 5). Recorded EC_50_ values for all isolates were >3800 µg/mL (Table 3). 

No differentiation between the S and W isolate groups nor between the isolates was discovered against dimethomorph for single concentrations, according to the Kruskal–Wallis test (*p* = 0.655), and for all of the concentrations together, according to discriminant function analysis. This fungicide was evaluated as completely ineffective against *P. cactorum* zoospore release, with EC_50_ values >5000 µg/mL.

Azoxystrobin is efficient against *P. cactorum* zoospore release at the concentration of 20 µg/mL. At medium concentrations (0.01–10.00 µg/mL), significant differences as high as 20% were found between groups S and W (*p* < 0.000). Considering all concentrations together, the differences were also significant (*p* = 0.002) (supplementary Table S8). On an individual level, one highly resistant isolate (EC_50_ = 140.60 µg/mL, 37.31% inhibition rate at a 20 µg/mL concentration) and one highly sensitive isolate (EC_50_ = 0.19 µg/mL, complete inhibition at 20 µg/mL) was found compared to the others (Table 3). Both of these isolates are members of the S group. The EC_50_ values and zoospore release inhibition rate are described in Table 3 and supplementary Table S7, respectively.

Fluopicolide was evaluated as effective against the zoospore release. Zoospores of all isolates of *P. cactorum* were almost completely inhibited at 10.0 µg/mL. The EC_50_ ranged between 0.1479 and 4.807, with a mean value of 0.731 µg/mL. Significant differences were observed from discriminant analysis between isolates on an individual level (*p* < 0.000) and also between the S and W groups (*p* < 0.000). Nevertheless, no unequivocally resistant isolate was found. On an individual level, the differences between isolates were significant at concentrations between 0.10 and 20 µg/mL (*p* < 0.000), while on a group level the differences between the S and W groups were prominent at the concentrations of 10 and 20 µg/mL (*p* < 0.000) (supplementary Table S9). The EC_50_ values and zoospore release rate of each isolate and fungicide are available in Table 3 and supplementary Table S7, respectively.

### 3.4. Discrimination between the S and W Groups, Considering All Fungicides in All Concentrations, DA

**Mycelial growth:** The mycelial growth inhibition rates at all concentrations of all tested fungicides were included in the discriminant analysis, in an effort to determine the ability of such a set-up of the discriminant model to differentiate between the S and W group isolates, and to find the fungicides that contribute the most to this differentiation. A strong ability to discriminate between these two groups would mean that each group has a different sensitivity to the tested fungicides. The discriminating ability of the model in such a set-up was strong, λ_W_ = 0.0631, *p* < 0.000, and the ability of the model increased if single isolates were considered instead of their host-specific groups: λ_W_ = 0.0000, *p* < 0.000. The fungicides contributing the most to such a differentiation were metalaxyl, dimethomorph and fluopicolide, respectively, although the remaining fungicides were also evaluated as significantly contributing. These results show that the relation of *P. cactorum* isolates to fungicides is to some extent predictable on the basis of their membership in defined groups. However, to an even greater extent, the sensitivity of isolates to a wider spectrum of fungicides seems to be one of their individual characteristics (supplementary Table S10).

**Sporangial formation:** The results of the discriminant analysis, based on the inhibition of sporangia formation at all concentrations of all four tested fungicides, together show a high differentiation between the S and W groups (λ_W_ = 0.1254; *p* < 0,000), and the discriminatory power of the model increased on the level of the individual isolates (λ_W_ = 0.0000; *p* < 0.000). All four tested fungicides were evaluated as contributing to the differentiation between these two groups (supplementary Table S11).

**Zoospore release:** The results of the discriminant analysis, based on the zoospore release inhibition at all concentrations of all fungicides, together show a moderate differentiation between the S and W groups (λ_W_ = 0.51854; *p* < 0.000), as well as on an individual level (λ_W_ = 0.00001; *p* < 0.000) (supplementary Table S12).

### 3.5. Discrimination between the S and W Groups Considering All Fungicides in All Concentrations—Discriminant Analysis Based on the Results of All Three Analyses

**Figure 3:**
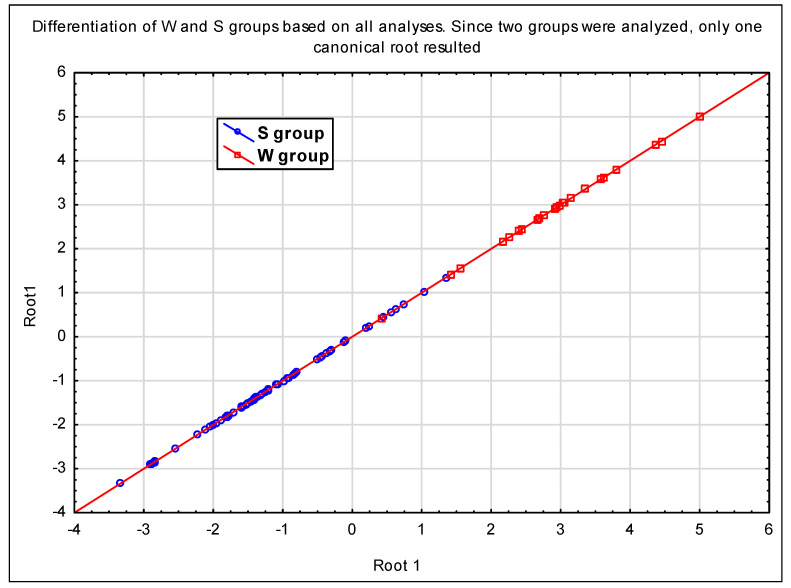
The differentiation of *P. cactorum* host-specific groups, documented by the canonical roots of discriminant analysis based on the results of all three analyses. S—isolates from strawberry plants and W—isolates from woody plants.

Discriminant analysis based on all of the performed experiments resulted in the values of λ_W_ expressing a differentiation between the S and W groups and between the *P. cactorum* isolates. The values of λ_W_ show that both models are substantially able to discriminate between the S and W groups (λ_W_ = 0.217; *p* < 0.000) or between the *P. cactorum* isolates (λ_W_ = 0.000; *p* < 0.000). Metalaxyl was evaluated as the most important fungicide in differentiating between the isolates (Table 5). Since only two groups of *P. cactorum* (S and W) were analyzed, only one canonical root was obtained as a result of the canonical part of the performed DA. This single root differentiates well between these two host-specific groups (Figure 3). This canonical root is highly correlated with the influence of metalaxyl in a mycelial growth inhibition and zoosporangia formation experiments, covering 0.427% of the total variability; the rest of the variability is associated with other fungicides.

The discriminant analysis based on the data from all of the assays also resulted in estimates of the Mahalanobis distances between isolates. The Mahalanobis distances matrix shows that the majority of isolates are quite similar in their aggregate response to the tested fungicides. In this matrix, as an important exception, primarily those isolates resistant to metalaxyl in the growth and zoosporangia formation assays are evaluated as differing from the majority. An association with the resistance to other fungicides was not obvious in this matrix. Another exception was the isolate CBS111725, differing from the majority at a rate similar to the metalaxyl-resistant ones, although the resistance to this fungicide was not evaluated as having significantly increased; still, this isolate was closer to the metalaxyl-resistant ones. This isolate, originating from Viburnum sp., is a member of the genetic lineage H [38], together with some of our other tested isolates (Table 1); however, none of the remaining isolates of this lineage, nor members of other lineages (C1 and F) included in the current study, differed substantially from the majority. The results of the Mahalanobis distances matrix are given in supplementary Table S13.

### 3.6. A Comparison of Similarity among Isolates Based on Their Response to Fungicides, with the Similarity Being Based on Genetic Similarity

The current use of some isolates that were also used in the previous study enabled us to compare their similarity based on the isolates’ response to fungicides, with the similarity being based on their already published genetic arrangement [38,39]. Analogies between these two similarity matrices, expressed in a percentage, are given in supplementary Table S14; a total summation of the similarities is given in Table 8. These values indicate that the isolates of the S group have closer genetic relations among each other than for those of the W group. The similarity in resistance between W group members more closely follows the relations based on the genetic matrix. The similarity between groups S and W is ca. 80% for both matrices. Inside the S group, the metalaxyl-resistant isolates were substantially different from the others while in the W group, an important dissimilarity was associated only with the isolate CBS111725, the only member of the genetic lineage H in the sense of the previous study [38].

## 4. Discussion

The genus *Phytophthora* includes the most significant plant pathogens worldwide, which endanger the supply of the most important crops such as potato, soya and many others [46]. The suppression of their infection on various crops mainly relies on the use of fungicides, and the production of some crops is unimaginable without their use [47]. Such a widespread use of these fungicides resulted in the rapid rise of resistance against them in various species of *Phytophthora*, soon after their commercial release [48,49]; the populations of many *Phytophthora* spp. include some number of genotypes resistant to various fungicides [49,50]. Our results also confirmed the presence of resistant strains of *P. cactorum* in Czech strawberry fields. Resistance against fungicides is distributed unevenly, and in some measure reflects the host-specific groups of *P. cactorum*; various fungicides are also unequally efficient against different life stages.

The most obvious is the variability of resistance against metalaxyl, which has been documented in the isolates we tested. The isolates are either fully sensitive or highly resistant, which is in accordance with the assumed monogenic nature of resistance to this fungicide [51,52]. In our analyses, only four isolates (19%) originating from strawberry plants in the Czech Republic (S) were resistant, while all of those from woody plants (W) were normally sensitive. Resistance against this fungicide is well documented in many *Phytophthora* spp. [53,54,55]. Two out of the four isolates resistant to metalaxyl that we found are also resistant to dimethomorph. These two multi-resistant isolates originate in the same locality, together with one isolate resistant only to dimethomorph and one isolate sensitive to both of these fungicides. The uneven distribution of resistance to metalaxyl could indicate the different origin of the isolates, as mentioned by [56] on *P. infestans*. The common presence of both resistant and sensitive isolates in one locality (Table 3) suggests either at least two independent introductions into the locality, or the rapid development of resistance in some of the strains present. Since not all of the isolates resistant against one of these two fungicides are also resistant against the second fungicide, their multidrug resistance does not match the cross-resistance, which is also ruled out by the different mode of action (discussed below) of these fungicides. The formation of resistance against dimethomorph has been considered improbable due to the polygenic nature of such resistance [57], although the dimethomorph-resistant mutants were acquired in laboratory conditions [58,59]. The results of current analyses show the resistance formation in *P. cactorum* isolates in field conditions, although the number of such events is unclear, considering at least two different types of isolates resistant to this fungicide are present together in one locality (18_07_2S12 is resistant to dimethomorph + metalaxyl; 18_07_6 is resistant only to dimethomorph). Both the dimethomorph- and metalaxyl-resistant isolates that originate from *P. cactorum* populations in strawberry fields in the Czech Republic, are considered to belong to the genetically homogenous lineage “S” [39]. The variability in response to these fungicides is somewhat inconsistent with the low genetic diversity in that group. 

Fluopicolide was evaluated as efficient against all of the life stages of *P. cactorum* we tested. We did not find any considerably resistant isolate, represented by some exceptionally high values of EC_50_, although resistance to this fungicide has already been described in various *Phytophthora* spp. [60,61,62]. Unlike the example of the two previous fungicides, sensitivity against fluopicolide did not differ substantially between the isolate groups S and W. However, two isolates of the W group were substantially more sensitive to this fungicide in comparison to the rest of the isolates, which evokes the question of which resistance level is common in *P. cactorum*. Except for one, all of the the *P. cactorum* isolates included in the study originated in Europe, where this species is assumed to be non-indigenous [1], and the origin of the *P. cactorum* species is unknown. Therefore, using an isolate from the natural population to determine the natural level of sensitivity was not possible. These natural levels of sensitivity expressed as EC_50_ values determined in wild populations of *P. capsici, P. nicotianae* and *P. erythroseptica* [43,44,45] were lower than 0.3 μg/mL, while the EC_50_ values of resistant mutants were on the level of units to hundreds of μg/mL [43]. The EC_50_ values we calculated for our *P. cactorum* isolates (ca. 0.7-4.0 μg/mL; Table 1) were somewhat larger in comparison to the values published for wild populations of the other *Phytophthora* spp. The resistance level of all *P. cactorum* isolates against fluopicolide was also evaluated by the estimated values of the resistance factors (RF; the rate of EC_50_ of a particular isolate to the EC_50_ of a strain from the wild population) [43], by comparing the calculated EC_50_ of *P. cactorum* to the known values of other mentioned *Phytophthora* spp. These RF values were between 2.0 and 66.7, which corresponds to intermediate resistance according to Lu et al. [43] and indicates an increase compared to the theoretical natural level. All but two *P. cactorum* isolates we tested had more or less increased resistance, which is not entirely consistent with the assumed low risk of resistance development against fluopicolide for *Phytophthora* spp. [45]; however, this fungicide is still able to inhibit the growth of *P. cactorum* isolates. 

The rest of the tested fungicides—azoxystrobin, fenamidone, cymoxanil and propamocarb—were evaluated as inefficient in the mycelial growth assay. Their effect was indistinguishable between the S and W isolate groups except for cymoxanil, whose effect was different for each of the two groups. Cymoxanil was found inefficient in mycelial growth inhibition, where only about 25% of mycelial growth was inhibited even at the concentration of 100 µg/mL, which is congruent with the results of other studies [63,64]. All of the isolates we tested were resistant, although substantial variation in the resistance of the isolates was observed on an individual level. Our evidence of a *P. cactorum* population highly resistant to cymoxanil is surprisingly in contrast to the sensitive behavior of the *P. cactorum* in Florida (USA), as reported by Marin et al. [27]. Such a high magnitude of sensitivity could be associated with rapid pathogen adaptation, the presence of diverse lineages in different parts of the world, or the distinct frequency of the application of this chemical [65,66,67]. The mode of action of cymoxanil is unknown [68]. The substantial differentiation between the responses of the S group isolates to cymoxanil, despite their close genetic relationship (supplementary Table S14), indicates the probable qualitative (monogenic) nature [69] of resistance to this fungicide, which enables such a rapid increase in resistance.

The efficiency of fungicides varies between the different life stages of a pathogen, and is probably determined by the various modes of action of the active ingredients. In the case of metalaxyl, the mycelial growth phase and sporangia formation are the stages affected by this fungicide, because metalaxyl inhibits the synthesis of ribosomal RNA by disrupting the activity of RNA polymerase [51,70,71]. Since the growth of hyphae is necessarily associated with RNA synthesis, metalaxyl significantly affects such regions but does not have an impact on zoospore release, probably because such a process is not directly determined by RNA synthesis. A similar principle could also be considered for the inefficiency of dimethomorph against zoospore release, although the mode of action of dimethomorph is different. This fungicide inhibits cell wall deposition in actively growing regions of hyphae [49,57,72], therefore it is efficient against mycelial growth or sporangia formation, but not against zoospore release. Azoxystrobin, which we showed to be the most effective against zoospore release, relies on the inhibition of the ATP supply by mitochondria. This is achieved by the disruption of the activity of cytochromes which determine oxidative respiration [73]. Since respiration is necessary for energetically demanding metabolic processes, such as active movement, zoospores are affected by this fungicide. However, mitochondria are also present in growing hyphal tips in high numbers, with a high probability that they do not respire or synthesize ATP there but play other roles [74], which could explain the inefficiency of azoxystrobin against mycelial growth. The only fungicide efficient against all three tested life stages of *P. cactorum*, fluopicolide, causes the disruption of cytoskeletal proteins such as actins, integrins, tubulins and spectrins [75]. These structures are necessary for all living cells, so their damage affects all of the life stages we tested.

Our analyses revealed a substantial differentiation between the host-specific groups of the *P. cactorum* isolates. The host specificity was revealed in this species of plant pathogen, where isolates originating from strawberry plants are significantly more virulent to this host than to others, or are even totally unable to infect other host species. Conversely, isolates from woody plants are unable to attack strawberry plants [4,18,76,77]. Finer structuring into genetic lineages was revealed, which is associated with the genetic background of this species [39]; this division also roughly respects the host-specific grouping. Such differentiation is also obvious on the level of resistance to fungicides, which is evidenced by the low λ_W total_ (0.22, *p* < 0.000, Table 5) in the DA, calculated on the basis of the collective data of all current assays. For some fungicides, such differentiation is also obvious separately in individual analyses. Although we identified the isolates which are totally resistant to some fungicides, and although they are usually members of the group S, the differences between the S and W groups were also perceptible if the obviously resistant isolates were considered as a separate group in the analysis (Table 4, Table 5, Table 6 and Table 7). The reason for such a differentiation could only be hypothesized as the result of a greater use of fungicides against *P. cactorum* in strawberry fields (for S group members) than in other types of land use (for W group members). The shift in resistance is documented by our comparison of a similarity matrix based on fungicides, with one based on genetic information. This comparison shows the decreased mutual average similarity of the isolates of the S group based on fungicides (75%) compared to their genetic similarity (93%). No similar shift is visible in the W group, where the average similarities are approximately equal (83% for genetics and 84% for fungicides; Table 8). The similarities among isolates of the S group based on fungicide resistance are lower (75%) than those among isolates of the W group (84%), although the S group members are mutually genetically closer (S—93%, W—83%) and the W group members originate from a significantly wider region. These numbers document a shift caused very probably by the frequent use of fungicides, since although some members of the W group originate from apple trees where fungicide use cannot not be excluded, their incomparably more intensive use in strawberry plantations can be assumed. However, as documented by the estimated value of the resistance factor [43] for fluopicolide, in relation to fungicides, even isolates of the group W do not behave like wild population strains [43,44,45]. Since *P. cactorum* is assumed not to be indigenous to Europe [18], the resistance of the entire European population could be influenced by the features of genotypes which were introduced in Europe.

We revealed the differences in resistance against fungicides on various levels, i.e., between host-specific groups, between diverse life stages and between isolates, and this should be carefully taken into consideration in the further use of these fungicides in plant protection. Of these generally efficient fungicides—metalaxyl, dimethomorph, azoxystrobin and fluopicolide—only the last one is useful against all stages of a pathogen. Although no isolate obviously resistant against fluopicolide was found, the entire population probably has a somewhat increased resistance against this fungicide. The differences between populations from strawberry and woody plants indicates the increased resistance against fungicides in the first mentioned group, which is most likely the result of the continual use of these fungicides. Even in the case of the careful use of fungicide rotation, some *P. cactorum* strains acquire resistance against the fungicides used, because some life stages are insensitive to a particular fungicide and survive the treatment. Similar accidental contacts of a pathogen with a non-lethal fungicide application gradually increase the resistance level of the population [78]. The use of anti-resistant strategies formulated to prevent the development of resistance [79] should be the necessary minimum basis for the sustainability of plant protection.

## Figures and Tables

**Table 1:** Isolates of *Phytophthora cactorum* used in the current study.

Isolate ID	GenBank Accession Numbers of ITS Region of rDNA	Membership of Isolates in Genetic Lineages	Country of Origin	Host (the Groups According to Host: S-Strawberry/W-Woody Plants)
17_03_23a	OM444198	S	Czech Republic	Strawberry (S)
17_03_5	OK257587	S	Czech Republic	Strawberry (S)
17_04_8	OK257597	S	Czech Republic	Strawberry (S)
17_07_12a	OK257601	S	Czech Republic	Strawberry (S)
17_08_17b	OK257604	S	Czech Republic	Strawberry (S)
17_09_12	OK257605	S	Czech Republic	Strawberry (S)
17_09_14a	OK257606	S	Czech Republic	Strawberry (S)
17_09_14b	OM444199	S	Czech Republic	Strawberry (S)
17_12_16	OK257618	S	Czech Republic	Strawberry (S)
17_12_18a	OK257620	S	Czech Republic	Strawberry (S)
17_12_27	OK257625	S	Czech Republic	Strawberry (S)
17_12_3	OK257610	S	Czech Republic	Strawberry (S)
17_15_1	OK257629	S	Czech Republic	Strawberry (S)
17_15_10b	OM444200	S	Czech Republic	Strawberry (S)
17_15_8	OK257631	S	Czech Republic	Strawberry (S)
17_26_14	OK257649	S	Czech Republic	Strawberry (S)
18_02_1b	MW193099	S	Czech Republic	Strawberry (S)
18_07_14	OK257669	S	Czech Republic	Strawberry (S)
18_07_2S12	ON490471	S	Czech Republic	Strawberry (S)
18_07_2S5	OM444201	S	Czech Republic	Strawberry (S)
18_07_6	OK257668	S	Czech Republic	Strawberry (S)
CBS111725	AB700469 *	H	the Netherlands	Viburnum (W)
ICMP11853	*	C1	New Zealand	Malus (W)
M5620	*	C1	Switzerland	Apple (W)
M5624	*	H	Switzerland	Apple (W)
M5652	*	C1	Switzerland	Apple (W)
M5654	LN907667 *	H	Switzerland	Apple (W)
Ph4	*	F	Finland	Betula (W)
Ph8	*	F	Finland	Betula (W)
PS-719	*	H	Spain	Aucuba (W)

Isolates marked by (*) have already been unequivocally identified as *P. cactorum* in the aforementioned studies.

**Table 2:** The tests performed on *Phytophthora cactorum* isolates at different concentrations of particular fungicides.

Tested Fungicides	Assays Performed	Tested Concentrations of Fungicides (µg/mL)				
		0.001, 0.010, 0.100, 1.000, 10.000	20.000	30.000	60.000	100.000
Azoxystrobin	GI	×				×
	SF	×				
	ZR	×	×			
Cymoxanil	GI	×				×
	SF					
	ZR					
Dimethomorph	GI	×	×	×		
	SF	×	×			
	ZR	×	×			×
Fenamidone	GI	×				×
	SF					
	ZR					
Fluopicolide	GI	×	×			
	SF	×				
	ZR	×	×			
Metalaxyl	GI	×	×		×	×
	SF	×	×			×
	ZR	×	×			×
Propamocarb	GI	×				×
	SF					
	ZR					

The assays performed for particular concentrations of the tested fungicides are marked by (×). Assays performed: GI—growth inhibition assay; SF—sporangia formation assay; ZR—zoospore release assay.

**Table 3:** EC_50_ values recorded for all *P. cactorum* isolates against tested fungicides in the mycelial growth inhibition, sporangia formation and zoospore release assays.

ID	Host (S/W)	Mycelial Growth Inhibition Assay							Sporangia Formation Assay				Zoospore Release Assay			
		Azoxystrobin	Cymoxanil	Dimethomorph	Fenamidone	Fluopicolide	Metalaxyl	Propamocarb	Azoxystrobin	Dimethomorph	Fluopicolide	Metalaxyl	Azoxystrobin	Dimethomorph	Fluopicolide	Metalaxyl
**17_03_23a**	**S**	195,914	1298	1.29	89.15	2.648	1.993	289,048	0.4655	0.1995	0.2739	0.05605	4.762	1,547,283	0.5874	712,245
**17_03_5**	**S**	312.6	215.1	0.9438	81.85	1.321	1,342,520,801	3,539,596	0.3532	0.295	0.3604	54110046	4.227	1,004,978	0.5652	44,694
**17_04_8**	**S**	504.3	12,390	0.6562	91.03	1.64	8,310,521,589	680,194	0.03881	0.5123	0.2633	66858235	1.186	16,867	0.3461	3,977,075
**17_07_12a**	**S**	105,236	231201	0.7062	121.7	1.448	0.1964	388,858	9.429	0.3631	0.4206	0.2329	2.847	9,549,762	0.421	1,707,215
**17_08_17b**	**S**	566.8	1885	0.6291	112.4	1.004	0.2858	2,890,512	0.6502	1.78	0.5914	0.1553	1.264	1,249,011	0.9721	3,839
**17_09_12**	**S**	113,959	62,938	0.4918	76.1	1.292	0.4249	1,877,567	0.5229	0.19	0.3158	0.05898	1.238	10,965,812	0.5307	181,985
**17_09_14a**	**S**	984,459	195,824	1.026	108.4	1.348	0.5504	167,983	0.5744	0.2165	0.2123	0.1929	1.773	19,160,204	4.807	1,245,070
**17_09_14b**	**S**	52,106	965,326	0.7184	112.7	1.554	1.104	614,730	1.536	0.1069	0.2016	0.1948	1.204	1,423,195	0.5533	286,405
**17_12_16**	**S**	470.9	330,189	1.228	114.6	2.647	0.3061	427,846	0.04895	0.07692	0.2677	0.258	0.8263	370,732	0.2777	326,305
**17_12_18a**	**S**	11,150	65,131	0.4643	105.1	1.318	0.3223	1,134,430	0.2253	0.3235	0.2405	0.5872	2.152	1,596,963	0.3327	47,271
**17_12_27**	**S**	1341	116,595	0.7796	96.21	1.435	0.1849	505,783	0.08326	0.1405	0.287	0.1001	2.191	828,281	0.2352	111,847
**17_12_3**	**S**	1949	11,859	0.7249	128.9	2.07	0.2294	119,878	8.448	0.2791	0.2323	0.122	1.115	8,241,254,881	0.4373	1,212,003
**17_15_1**	**S**	1395	91,361	0.6589	89.12	1.23	0.358	186,731	0.6122	0.0961	0.3586	0.217	1.103	61,599	0.4456	39,810,861
**17_15_10b**	**S**	884.4	29,823	0.3218	79.7	1.421	1.23	630,081	0.3941	0.1099	0.242	0.2287	2.587	399,993	1.411	108,484
**17_15_8**	**S**	469.3	98,856	0.7015	124.2	1.203	0.1592	197,276	0.257	0.3373	0.2491	0.1895	4.817	233,912	1.767	723,065
**17_26_14**	**S**	3405	942.1	0.6608	111.7	1.565	0.5428	5,429,390	0.1585	0.1983	0.6331	0.3594	3.996	1,106,716	0.2193	1,019,196
**18_02_1b**	**S**	785.9	794.3	0.7407	109.5	0.8949	0.344	601,843	0.4343	0.3475	0.2518	0.1329	2.35	1,756,455	0.7535	123,113
**18_07_14**	**S**	2741	211,659	0.7205	114.5	1.16	0.2932	94281	0.7243	0.09996	0.2752	0.183	1.162	336,540	0.3259	400,499
**18_07_2S12**	**S**	331.4	137,569	11.32	76.44	1.338	8,815,969,175	194,938	0.3927	0.863	0.2556	279217	140.6	82,269	0.8776	3,351,129
**18_07_2S5**	**S**	306.4	3,196,697	12.52	119.8	1.622	4,755,197	1,619,544	0.07639	0.4521	0.173	437155	3.499	2,420,187	0.2512	857,285
**18_07_6**	**S**	4198	244,389	11.69	82.41	1.041	1.145	2,411,837	0.762	0.4636	0.3324	0.7482	0.1984	100,362	2.871	1,050,826
**CBS111725**	**W**	432.3	21,810	0.6382	80.18	0.7048	28.5	513,797	0.6084	0.2235	0.1231	1.175	4.418	29,978	0.2168	1,763,676
**ICMP11853**	**W**	1512	50,889	0.3776	84.54	1.918	0.03336	106,435	0.7824	0.3698	0.1277	1.729	3.976	425,167	0.382	5,046,614
**M5620**	**W**	315.7	27,081	0.1314	109.3	1.421	0.06669	150,248	65628	0.286	0.1066	2.894	8.972	1,384,175	0.1479	74,350
**M5624**	**W**	5418	2547	0.5169	115.8	3.999	0.08128	2,964,106	0.6467	0.8402	0.1953	0.952	2.978	160,204	0.3749	234,053
**M5652**	**W**	1,042,469	32,584	0.3393	78.42	1.921	0.05454	903,897	6.066	0.3587	0.198	0.7247	4.99	2,373,393	0.4813	2,781,817
**M5654**	**W**	351.1	30,261	0.3692	110.1	0.7094	0.0813	1,358,413	0.7182	0.2444	0.1917	2.824	4.941	67,014	0.5247	99,123
**Ph4**	**W**	1008	701,905	0.2968	95.2	0.7606	0.1013	540,871	0.4858	0.1585	0.212	1.449	4.979	5,969	0.3221	85,127
**Ph8**	**W**	215,330	18,289	0.5227	81.61	1.795	0.06541	1,122,558	1.128	0.1453	0.2328	1.053	8.947	6,801,140	0.3208	1,164,430
**PS_719**	**W**	13,146	23,786,913	0.6619	369.6	1.758	0.4167	424,008	0.81	0.2129	0.2238	0.9651	10.3	219,442	0.1947	69,265

**Table 4:** Results of a multiple comparison of *p*-values of the Kruskal–Wallis test of *P. cactorum* growth inhibition caused by metalaxyl.

Metalaxyl Concentration (µg/mL)	Isolates Group Based on Host Specificity to Host Types (W/S)		Isolates Group Based on Host Specificity to Host Types (W/S)			Average Inhibition Rate (%)
			Multiple Comparison of *p*-Values			
			S	SRM	W	
**0.001**	**S**			0.790	1.000	1.245
	SRM		0.790		1.000	1.486
	W		1.000	1.000		1.244
0.010	S			**0.001**	0.679	4.056
	SRM		**0.001**		**0.000**	2.351
	W		0.679	**0.000**		5.749
0.100	S			**0.000**	**0.000**	20.558
	SRM		**0.000**		**0.000**	3.075
	W		**0.000**	**0.000**		55.896
1.000	S			**0.000**	**0.010**	69.869
	SRM		**0.000**		**0.000**	4.603
	W		**0.010**	**0.000**		76.476
10.000	S			**0.000**	1.000	90.713
	SRM		**0.000**		**0.000**	4.808
	W		1.000	**0.000**		89.581
20.000	S			**0.000**	1.000	99.697
	SRM	**0.000**			**0.000**	5.969
	W	1.000		**0.000**		91.924
60.000	S			**0.000**	0.969	100.000
	SRM	**0.000**			**0.000**	6.937
	W	0.969		**0.000**		97.731
100.000	S			**0.000**	0.969	100.000
	SRM	**0.000**			**0.000**	7.744
	W	0.969		**0.000**		99.018
All concentrations together	S			**0.000**	1.000	**-**
	SRM	**0.000**			**0.000**	**-**
	W	1.000		**0.000**		**-**

All tested isolates were organized into groups according to the host plant they were isolated from: S—strawberry, W—woody hosts. The group marked as SRM was created by three isolates originating from strawberry plants which are obviously resistant to metalaxyl.

**Table 5:** Results of discriminant analysis of all performed assays for differentiation between S and W groups of *P. cactorum* isolates, based on in-vitro responses of isolates against the tested fungicides.

Assay	Fungicide	Grouping on the Level between Host-Specific Isolates, Groups S/W		Grouping on the Level of Individual Isolates		Common Efficiency of the Fungicide against *P. cactorum*. Efficient (E)/Inefficient (I)
		Partial Wilk’s Λ	*p*-Value	Partial Wilk’s Λ	*p*-Value	
**Sporangia formation**	Metalaxyl	**0.301**	**0.000**	**0.000**	**0.000**	E
	Dimethomorph	**0.238**	**0.010**	**0.000**	**0.000**	E
	Fluopicolide	**0.279**	**0.000**	**0.000**	**0.002**	E
	Azoxystrobin	0.226	0.092	**0.000**	**0.000**	E
**Growth inhibition**	Metalaxyl	**0.256**	**0.001**	**0.000**	**0.000**	E
	Dimethomorph	**0.238**	**0.009**	**0.000**	**0.000**	E
	Azoxystrobin	0.217	0.832	**0.000**	**0.000**	I
	Fluopicolide	**0.231**	**0.036**	**0.000**	**0.000**	E
	Propamocarb	0.220	0.291	**0.000**	**0.000**	I
	Cymoxanil	**0.234**	**0.020**	**0.000**	**0.000**	I
	Fenamidone	0.220	0.282	**0.000**	**0.000**	I
**Zoospore release**	Azoxystrobin	0.225	0.112	**0.000**	**0.000**	E
	Dimethomorph	0.217	0.971	**0.000**	0.854	I
	Metalaxyl	0.217	0.926	**0.000**	0.085	I
	Fluopicolide	**0.248**	**0.002**	**0.000**	**0.000**	E
**Wilk’s Λ total**		**0.217**	**0.000**	**0.000**	**0.000**	**/**

The values of Wilk’s Λ and corresponding *p*-values are given for each fungicide in a particular assay, and also for the analysis including all partial assays. The values of Wilk’s Λ are on a scale between 0 and 1, where 0 = the best discriminating ability of the statistical model, and 1 = the zero discriminating ability of the model. The values of Partial Wilk’s Λ express the decrease in total Wilk’s Λ (in comparison to the total one) when a corresponding variable is removed from the model. This value expresses the importance of each variable for the discriminating ability of the model, where a large increase in the value comparing to the total Wilks Λ means a high importance, while a zero change means a low importance of the particular variable. The statistically significant values are marked in bold.

**Table 6:** Results of a multiple comparison of *p*-values of the Kruskal–Wallis test of *P. cactorum* growth inhibition caused by dimethomorph.

Dimethomorph Concentration (µg/mL)	Isolates Group Based on Host Specificity to Host Types (W/S)	Isolates Group Based on Host Specificity to Host Types (W/S)			Average Inhibition Rate (%)
		Multiple Comparison of *p*-Values			
		S	SRD	W	
0.001	S		**0.001**	**0.006**	2.012
	SRD	**0.001**		0.347	1.009
	W	**0.006**	0.347		1.329
0.010	S		**0.000**	**0.002**	3.906
	SRD	**0.000**		0.402	2.353
	W	**0.002**	0.402		2.641
0.100	S		**0.003**	**0.019**	8.377
	SRD	**0.003**		**0.000**	3.884
	W	**0.019**	**0.000**		13.963
1.000	S		**0.000**	**0.000**	60.500
	SRD	**0.000**		**0.000**	4.819
	W	**0.000**	**0.000**		77.875
10.000	S		**0.000**	0.900	99.999
	SRD	**0.000**		**0.000**	45.471
	W	0.900	**0.000**		100.000
20.000	S		0.000	1.000	100.000
	SRD	**0.000**		**0.000**	66.116
	W	1.000	**0.000**		100.000
30.000	S		**0.000**	1.000	100.000
	SRD	**0.000**		**0.000**	75.647
	W	1.000	**0.000**		100.000
All concentrations together	S		**0.000**	1.000	**-**
	SRD	**0.000**		**0.000**	**-**
	W	1.000	**0.000**		**-**

**Table 7:** Results of a multiple comparison of *p*-values of the Kruskal–Wallis test of *P. cactorum* growth inhibition caused by fluopicolide.

Fluopicolide Concentration (µg/mL)	Isolates Group Based on Host Specificity to Host Types (W/S/WSF)	Isolates Group Based on Host Specificity to Host Types (W/S)			Average Inhibition Rate (%)
		Multiple Comparison of *p*-Values			
		S	W	WSF	
0.001	S		1.000	0.982	1.367
0.001	W	1.000		1.000	1.297
0.001	WSF	0.982	1.000		1.095
0.010	S		1.000	0.417	2.674
0.010	W	1.000		1.000	2.556
0.010	WSF	0.417	1.000		2.250
0.100	S		0.224	**0.001**	3.909
0.100	W	0.224		0.047	4.698
0.100	WSF	**0.001**	0.047		5.396
1.000	S		**0.000**	**0.000**	46.373
1.000	W	**0.000**		**0.000**	39.315
1.000	WSF	**0.000**	**0.000**		61.044
10.000	S		0.896	**0.000**	75.586
10.000	W	0.896		**0.000**	77.007
10.000	WSF	**0.000**	**0.000**		99.160
20.000	S		1.000	**0.000**	97.582
20.000	W	1.000		**0.000**	98.004
20.000	WSF	**0.000**	**0.000**		100.000

All tested isolates were organized into groups according to the host plant they were isolated from: S—strawberry, W—woody hosts, WSF—woody host isolates sensitive to fluopicolide. The group marked as WSF was created by isolates originating from woody hosts, which are obviously more sensitive to fluopicolide in comparison to others.

**Table 8:** Combined average similarity in group S and W and among both groups.

Average Similarity Estimated for	Similarity Based on Genetics (%)	Similarity Based on Fungicides (%)	Analogy between Matrices (%)
Inside the group W	82.80	84.06	83.75
Inside the group S	93.19	74.93	72.49
Among the W and S groups	78.32	81.58	76.91

## Data Availability

Not applicable.

## Supplementary Materials

The following supporting information can be downloaded at: https://www.mdpi.com/article/10.3390/jof8101039/s1; Table S1: Mycelial Growth inhibition rate in (%) for each isolate against tested active ingredients of fungicides at different concentrations; Table S2: Sporangia formation inhibition rate (%) for each isolate tested against active ingredients of fungicides; Table S3: Results of Kruskall-Wallis test of sporangia formation inhibition by metalaxyl; Table S4: Results of Kruskall-Wallis test of sporangia formation inhibition by fluopicolide; Table S5: Results of Kruskall-Wallis test of sporangia formation inhibition by azoxystrobin; Table S6: Isolates group based on host specificity to host types (W/S); Table S7: Zoospores release inhibition rate (%) for each isolate tested against active ingredients of fungicides at various concentrations; Table S8: Results of Kruskall-Wallis test of zoospore release assay by azoxystrobin; Table S9: Isolates group based on host specificity to host types (W/S); Table S10: Results of Discriminant function analysis performed for mycelial growth inhibition assay for differentiation of S and W groups and among individual isolates on the basis of all tested active ingredients of fungicides at all concentrations; Table S11: Results of Discriminant function analysis performed for sporangia formation assay for differentiation of S and W groups and among individual isolates on the basis of all tested active ingredients oof fungicides at all concentrations; Table S12: Results of Discriminant function analysis performed for zoospore release assay for differentiation of S and W groups and among individual isolates on the basis of all tested active ingredients at all concentrations; Table S13: Similarity matrix based on all assays with fungicides; Table S14: Similarities between matrices based on Genetic similarity and Resistance similarity.
